# A Microfluidic Aptamer-Based Sensor for Detection of Mercury(II) and Lead(II) Ions in Water

**DOI:** 10.3390/mi12111283

**Published:** 2021-10-21

**Authors:** Wei-Hao Huang, Van-Phung Mai, Ruo-Yin Wu, Ko-Li Yeh, Ruey-Jen Yang

**Affiliations:** Department of Engineering Science, National Cheng Kung University, Tainan 70101, Taiwan; bok70229@gmail.com (W.-H.H.); phungbk.ksclc@gmail.com (V.-P.M.); lovelybabyoh1997@gmail.com (R.-Y.W.); leo5527tw@gmail.com (K.-L.Y.)

**Keywords:** aptamer, fluorescence resonance energy transfer, heavy metal ions, graphene oxide, microfluidic device, sensor

## Abstract

Heavy metal contaminants have serious consequences for the environment and human health. Consequently, effective methods for detecting their presence, particularly in water and food, are urgently required. Accordingly, the present study proposes a sensor capable of detecting mercury Hg(II) and lead Pb(II) ions simultaneously, using graphene oxide (GO) as a quenching agent and an aptamer solution as a reagent. In the proposed device, the aptamer sequences are labeled by FAM and HEX fluorescent dyes, respectively, and are mixed well with 500 ppm GO solution before injection into one inlet of the microchannel, and the heavy metal sample solution is injected into another inlet. The presence of Hg(II) and Pb(II) ions is then detected by measuring the change in the fluorescence intensity of the GO/aptamer suspension as the aptamer molecules undergo fluorescence resonance energy transfer (FRET). The selectivity of these two ions is also shown to be clear among other mixed heavy metal ions. The experimental results show that the aptamer sensors have a linear range of 10~250 nM (i.e., 2.0~50 ppb) for Hg(II) ions and 10~100 nM (i.e., 2.1~20.7 ppb) for Pb(II) ions. Furthermore, the limit of detection is around 0.70 ppb and 0.53 ppb for Hg(II) and Pb(II), respectively, which is lower than the maximum limits of 6 ppb and 10 ppb prescribed by the World Health Organization (WHO) for Hg(II) and Pb(II) in drinking water, respectively.

## 1. Introduction

Pollution caused by human activity is a serious worldwide problem nowadays, with massive environmental, financial and health ramifications. Among the many different types of contaminants found in water, land and the air, heavy metal ions pose a particularly significant risk to both the environment and human health. Consequently, effective methods for detecting trace amounts of heavy metal ions with high sensitivity and good selectivity are urgently required [1]. Mercury (Hg) is one of the most toxic heavy metals in common use and can cause a wide variety of unpleasant and dangerous disorders, including lung damage, diarrhea, nausea and permanent organ damage [2]. Lead (Pb) is also an extremely common heavy metal and is used extensively throughout the construction, water, electrical and battery industries. However, the presence of lead in water is harmful to human health even at low exposure levels and can result in delays in physical and mental development, together with serious attention and learning deficits [3,4].

During the past few years, many methods using different instruments have been reported for mercury and lead ion detection, including inductively coupled plasma mass spectrometry (ICP-MS) [5,6], cold-vapor atomic fluorescence spectroscopy (CV-AFS) [7], high-performance liquid chromatography (HPLC) [8], colorimetric sensors [9], magnetic beads [10], nanopore sensors [11,12], surface-enhanced Raman scattering (SERS) [13], electrochemical sensors [14,15] and aptasensors [16,17]. Although these methods provide reliable detection for mercury and lead ions, the mentioned methods suffer from some limitations. ICP-MS, CV-AFS and HPLC are costly and very time-consuming in pre-treatment [18,19]. Colorimetric sensors are relatively slow and have limited sensitivity [20]. Electrochemical sensors require modified electrodes, resulting in a complicated and time-consuming pre-treatment process [21,22]. Nanopore sensors can be fabricated by many different techniques. Therefore, the characteristic of the sensors depends on ionic conductance, the pore radius and surface charge effects, which may yield different detection results [23,24]. Among the various techniques that have been proposed, aptasensors are particularly attractive due to their high affinity, high specificity and wide versatility as a biological receptor for many different binding targets [25,26]. Furthermore, aptamers can be easily mass-produced, purified and tailored to the capture of particular targets through the design of specific sequences [26]. For example, aptamers with a thymine (T)-rich sequence can be used to form a mismatched complex (T-Hg^2+^-T) with Hg^2+^ ions by changing the configuration to a hairpin shape [27]. Similarly, aptamers with a guanine (G)-rich sequence can be made to readily bond with Pb^2+^ ions to form a G-quadruplex structure [28]. Aptasensors are compatible with many standard detection methods, including colorimetric, fluorescent and electrochemical signals.

Recent studies in aptamer sensors have focused increasingly on the use of attenuated total reflection surface-enhanced infrared absorption spectroscopy (ATR-SEIRAS) as an alternative sensing technique [29]. Fluorescent sensors have many practical advantages as a sensing tool, including ease of operation, high sensitivity and easily detectable signals. Most fluorescent sensors are based on the fluorescence resonance energy transfer (FRET) effect [30], in which energy transfer occurs between an excited donor fluorophore and an acceptor via a non-radiative mechanism without the absorption or emission of photons [31]. Graphene and graphene oxide (GO) have attracted growing interest in the sensing field in recent years, since aromatic ring structures based on graphene and GO surfaces readily adsorb and combine with many biological molecules (including aptamers) through a π-π stacking mechanism and electron transfer. The resulting aromatic ring structures thus provide an outstanding platform for fluorescence-based sensing [32,33,34,35]. Notably, graphene and its derivatives are beneficial not only in preventing the aptamers from changing their configuration before binding to the target, but also serve as excellent quenchers with minimal background interference. Thus, GO/aptamer sensors have unparalleled advantages for FRET-based sensing in many clinical, environmental and food testing applications nowadays [36,37]. For example, Zhao et al. reported a limit of detection (LOD) for Pb^2+^ detection of 300 pM using a GO/aptamer sensor [38]. Shi et al. showed a highly sensitive Pb^2+^ sensor with an LOD of 0.1 nM based on fluorescence quenching between GO and gold nanoparticles [39]. Lu et al. developed GO/aptamer sensors for detecting Hg^2+^, Pb^2+^ and Ag^+^ simultaneously; the LODs for Hg^2+^, Pb^2+^ and Ag^+^ were 0.2, 0.5 and 2 nM, respectively [40]. GO/aptamer sensors still pose challenges, such as (1) the small scale of the target causes huge steric hindrance in combining with the aptamer [41] and (2) a non-uniform GO material (size or shape) may limit their application [42]. The above results were collected either by expensive instruments or required a long time period. Therefore, we propose to design a device that possesses many of the advantages of microfluidics, namely a simple fabrication process, high throughput, ease of operation, low cost, good selectivity and low required volumes of sample and reagents. The details of the device will be given later.

**Figure 1:**
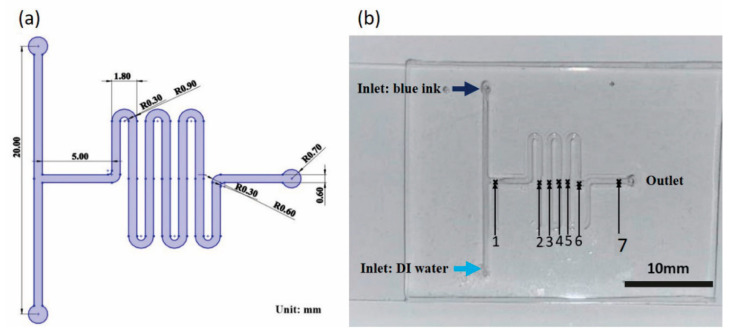
(**a**) Schematic illustration showing microfluidic device design and (**b**) photograph of actual microfluidic device.

In this study, we employ micro-machining technology (a type of computer numerical control (CNC) machining process) due to its low cost and the ability to manufacture complex molding structures [43]. The fabricated device has the form of a T-type micro-mixer incorporating a tortuous flow channel to reduce the distance between the inlet and the outlet and enhance the mixing efficiency. Furthermore, the mixer is fabricated by pouring and curing PDMS on a master mold to avoid the presence of residues after cleaning and to improve the repeatability of the experimental results accordingly. In the sensing process, a mixed GO/fluorescent-labeled aptamer solution is injected into the lower inlet (inlet 2) of the device, while a solution containing Hg^2+^ or Pb^2+^ ions is injected into the upper inlet (inlet 1) (see Figure 1). During the subsequent mixing process, the aptamers bond with the Hg^2+^ or Pb^2+^ ions to form thymine (T) or guanine (G) complexes, respectively, and the concentration of the ions is detected by observing the corresponding FRET-induced change in the fluorescence intensity [27,28,44]. Finally, the detection results are compared with those obtained from a commercial instrument—inductively coupled plasma mass spectrometry (ICP-MS).

## 2. Materials and Methods

### 2.1. Materials and Instruments

Graphene oxide (GO) dispersion was purchased from Graphenea Inc. (San Sebastián, Spain); the radial size of the GO sheet was below 10 μm. Nitric acid (HNO_3_), mercury(II) nitrate (Hg(NO_3_)_2_) and lead(II) nitrate (Pb(NO_3_)_2_) were purchased from Nihon Shiyaku Industries Ltd. (Taipei, Taiwan). The other chemicals used in the study—MgCl_2_, Na_2_HPO_4_, KH_2_PO_4_, HCl, NaOH—were obtained from Sigma-Aldrich Co (St. Louis, MO, USA); NaCl, KCl and CuSO_4_ were obtained from J.T. Baker Chemical Co (Radnor, PA, USA); CaCl_2_ and CoCl_2_ were obtained from Panreac (Barcelona, Spain). Poly-dimethylsiloxane (PDMS) was purchased from Sil-More Industrial Ltd. (Taipei, Taiwan). The solvents were prepared using ultrapure water (18.2 MΩ). All of the chemicals were of analytical grade and were used as received, without further purification. Two DNA probes modified with FAM and HEX fluorescent dye at the 3’ end, respectively, were purchased from Protech Technology Enterprise Co., Ltd. (Taipei, Taiwan). Aptamer solutions were prepared by dissolving the DNA probes in 100 mM of PBS (pH 7.4). The sequences of the two aptamer probes were as follows:

Probe for Hg^2+^: 5′-TTCTTTCTTCGCGTTGTTTGTT-FAM-3′

Probe for Pb^2+^: 5′-GGAAGGTGTGGAAGG-HEX-3′

The microfluidic device was fabricated using an EGX-400 Professional Rotary Engraver (Roland, Shizuoka, Japan) and a PDMS replication process. The mixing efficiency was evaluated using an ECLIPSE Ti Inverted research microscope (Nikon, Tokyo, Japan). In addition, the fluorescence measurements were obtained using a LSM780 scanning laser confocal microscope (Zeiss, Jena, Germany). The excitation wavelength of FAM and HEX fluorescent dye was 495 nm and 535 nm, respectively. The corresponding emission wavelength of the fluorescence intensity was measured at 520 nm and 556 nm, respectively.

### 2.2. Evaluation of Mixing Efficiency

Mixing between two fluids is an essential step in microfluidic devices for enhancing different species mixed and their subsequent reaction. There are many techniques available to achieve such purposes proposed in the literature [45,46,47,48]. We designed a simple passive mixer with a cured flow path in this study. Figure 1a illustrates the geometry of the microfluidic channel in the proposed sensing device. A mold was first fabricated in PMMA using the EGX-400 engraver and a replication process was then performed to transfer the microchannel to a PDMS substrate. Finally, the substrate was bonded to a glass slide using oxygen plasma treatment to form the final microfluidic assembly (see Figure 1b). The microchannel had two inlets, each one had a 10 mm length, and the distance of the tortuous microchannel from location #1 to the outlet was 87 mm. The channel width and height were 0.6 mm and 0.15 mm, respectively.

The mixing performance of the device was illustrated by injecting blue ink and DI water into the upper and lower inlets, respectively, and then calculating the mixing efficiency at each of the seven locations marked in Figure 1b in accordance with

(1) $$\mathrm{Mixing}\;\mathrm{Index}\;\left(\mathrm{MI}\right)=1-\sqrt{\frac{1}{N}\sum_{i=1}^{N}{\left(\frac{I_{i}-I_{\mathrm{mean}}}{I_{\mathrm{mean}}}\right)}^{2}},$$

where I_i_ is the intensity value of pixel i, I_mean_ is the mean intensity of the initial images prior to mixing and N is the total number of pixels within the captured image [45,49,50]. The value of I_i_ was analyzed by ImageJ software [51].

The flow field and the associated concentration of the two fluids in the 3D microchannel were also simulated by solving the Navier–Stokes equations coupled with the Nernst–Planck equations using commercial software, COMSOL Multiphysics (version 5.4, COMSOL Inc., Burlington, MA, USA). The mixing index (*MI*) was calculated numerically by the following integration:

(2) $$MI=1-\frac{\int_{A}\left|C-C_{i}\right|dA}{\int_{A}\left|C_{0}-C_{i}\right|dA},$$

where *C* is the concentration at a point on a cross-section plane *A*, *C_i_* is the concentration under the completed mixing state (i.e., *C_i_* = (1 + 0)/2 = 0.5, value 1 and 0 is the concentration at upper and lower inlet, respectively), and *C*_0_ is the concentration at the inlet (we choose *C*_0_ = 1 in the calculation).

### 2.3. Evaluation of Quenching Efficiency

The fluorescence quenching efficiency of the GO/aptamer solutions is strongly dependent on the relative concentration of GO. Thus, a series of experiments was performed to measure the fluorescence intensity change in the fluorescent-labeled aptamer solutions (100 nM) with different concentrations of GO in a glass-bottom dish. For each sample, the quenching efficiency was computed as (f_0_ − f)/f_0_, where f_0_ is the original aptamer fluorescence intensity and **f** is the measured fluorescence intensity in the presence of both aptamer and GO.

### 2.4. Experimental Setup

**Figure 2:**
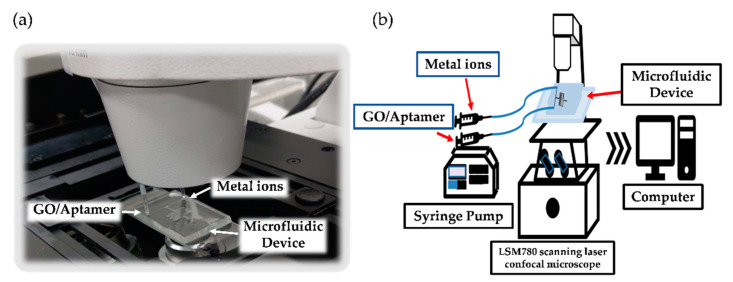
(**a**) Experimental setup used for Hg^2+^ and Pb^2+^ ion detection. (**b**) Schematic integration of the measurement system.

Figure 2 shows the schematic of the experimental settings. The metal ion solutions and GO/aptamer suspensions were injected into the upper and lower inlet channels of the microfluidic device, respectively. The resulting change in the fluorescence intensity of the GO/aptamer suspension was observed at Point #7 in the microfluidic channel (see Figure 1b) and the quenching effect was then evaluated by image analysis software installed on an interfaced PC in accordance with (F − F_0_)/F, where F is the measured fluorescence intensities at the observed point [44]. F_0_ is the fluorescence intensity when fully quenched by GO and its ideal value is 0.

## 3. Results

### 3.1. Mixing Performance of Microfluidic Device and Quenching Efficiency of GO/Aptamer Suspensions

**Figure 3:**
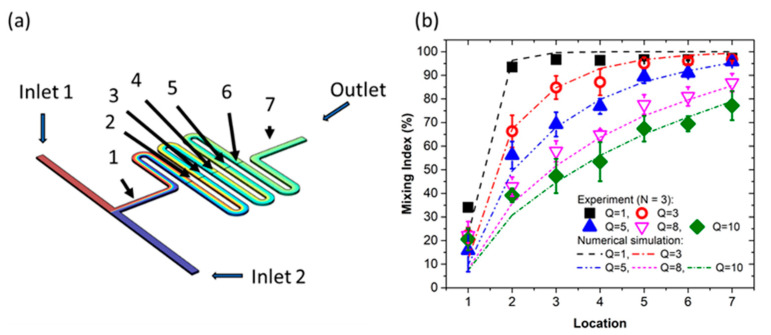
(**a**) Concentration contour distribution via numerical simulation. (**b**) Mixing index (MI) at different locations along the microchannel as function of flow rate (Q: μL/min). Experimental and numerical simulation results mutually agree well. Over 95% mixing index can be obtained for a flow rate lower than 5 μL/min at detection location #7.

Figure 3a shows a top view of the simulated concentration contour along the microchannel. Figure 3b shows the mixing index (MI) at different locations of the microchannel as a function of the flow rate (Q: μL/min). Each experimental datum was repeatedly measured three times (N = 3). The numerical results were shown to be consistent with the experimental results. The results show that the lower flow rates (i.e., below 5 μL/min) had better mixing indices at location #7, where the MI was over 95%. The reason is that the fluid requires more time to travel through the microchannel for a lower flow rate and the contact/diffusion time between the two fluids increases. Therefore, all subsequent experiments conducted in this study adopted the flow rate of 5 μL/min to ensure that the two fluids were well mixed.

**Figure 4:**
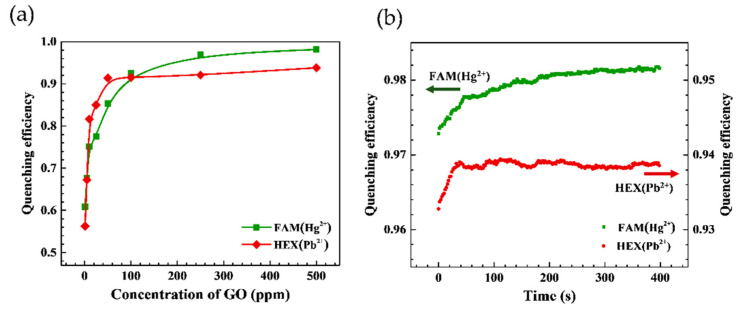
(**a**) Quenching efficiency for suspensions consisting of FAM(Hg^2+^)- and HEX(Pb^2+^)-labeled 100 nM aptamer and GO solution with concentration ranging from 1 to 500 ppm, and (**b**) long-term quenching efficiency of suspensions of FAM(Hg^2+^)- and HEX(Pb^2+^)-labeled aptamers and GO solution with concentration of 500 ppm.

The quenching efficiency of the GO/aptamer suspensions was evaluated for various GO concentrations. The results presented in Figure 4a show that for both aptamer probes (i.e., FAM(Hg^2+^)- and HEX(Pb^2+^)-labeled probes), the quenching efficiency remains approximately constant as the GO concentration is increased beyond 100 ppm. Thus, to ensure the complete suppression of the GO concentration effect, an excess concentration of 500 ppm GO was used in all of the remaining experiments, unless stated otherwise [52]. The long-term quenching effect of the GO/aptamer suspensions was observed in glass-bottom dishes over a period of 400 s. As shown in Figure 4b, the fluorescence intensity of both probes gradually reduced over time as a result of the FRET GO quenching effect. From inspection, the quenching efficiencies of the FAM(Hg^2+^)- and HEX(Pb^2+^)-labeled probes stabilized at approximately 0.98 and 0.94 around 100 s, respectively.

### 3.2. Sensitivity of Hg^2+^ and Pb^2+^ Detection

**Figure 5:**
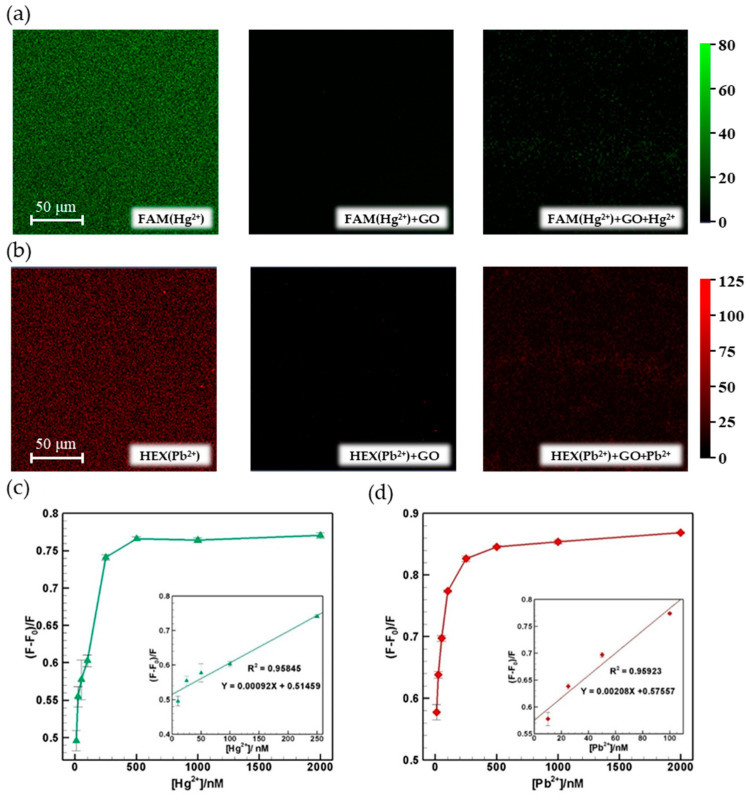
(**a**) Fluorescence images of FAM(Hg^2+^)-labeled probes and (**b**) fluorescence images of HEX(Pb^2+^)-labeled probes. The left, center and right images are the origin probes, quenched probes and probes restored by the target ion, respectively. The images were captured by a scanning laser confocal microscope using 20× magnification. (**c**) Fluorescence intensity recovery following mixing with Hg^2+^ solutions with concentrations ranging from 10 to 2000 nM (500 ppm GO). Insert: calibration curve for Hg^2+^ solutions with concentrations ranging from 10 to 250 nM, and (**d**) fluorescence intensity recovery following mixing with Pb^2+^ solutions with concentrations ranging from 10 to 2000 nM (500 ppm GO). Insert: calibration curve for Pb^2+^ solutions with concentrations ranging from 10 to 100 nM.

FAM(Hg^2+^)- and HEX(Pb^2+^)-labeled aptamers with a concentration of 100 nM were mixed with 500 ppm GO solution and incubated for 10 min to ensure the complete quenching of the aptamers. It should be noted that the quenching efficiencies for FAM(Hg^2+^-/HEX(Pb^2+^)-labeled probe can reach over 90% around 40 s/80 s, respectively. The suspensions were then injected into the lower inlet of the microfluidic device. Meanwhile, Hg^2+^ samples with concentrations of 10, 25, 50, 100, 250, 500, 1000 and 2000 nM (approximately 2.0, 5.0, 10.0, 20.1, 50.2, 100.3, 200.6 and 401.2 ppb) and Pb^2+^ samples with concentrations of 10, 25, 50, 100, 250, 500, 1000 and 2000 nM (approximately 2.1, 5.2, 10.4, 20.7, 51.8, 103.6, 207.2 and 414.4 ppb) were injected into the upper inlet of the device. The travelling time for both fluids from the inlets to the outlet was around 90 s at 5 μL/min. For each injected sample, the resulting change (i.e., increase) in the fluorescence intensity was recorded at location #7 in the microfluidic channel after 180 s to ensure that the flow field was stabilized. Figure 5a,b show the fluorescence images of the FRET process for the FAM(Hg^2+^)- and HEX(Pb^2+^)-labeled probes within the microchannel at location #7, respectively. As the sample solution flowed along the tortuous microchannel, Hg^2+^ or Pb^2+^ ions mixed with the quenched solution; then, the FRET effect restored the fluorescence intensity at location #7. The corresponding results are presented in Figure 5c,d for the FAM(Hg^2+^)- and HEX(Pb^2+^)-labeled probes, respectively. For both probes, the fluorescence intensity remained approximately constant as the metal ion content increased beyond 500 nM. However, the fluorescence intensity increased linearly with the Hg^2+^ concentration from 10 to 250 nM (R^2^ = 0.95845) and with the Pb^2+^ concentration from 10 to 100 nM (R^2^ = 0.95923). The corresponding correlation relationships have the form of Y = 0.00092X + 0.51459 and Y = 0.00208X + 0.57557, respectively.

**Figure 6:**
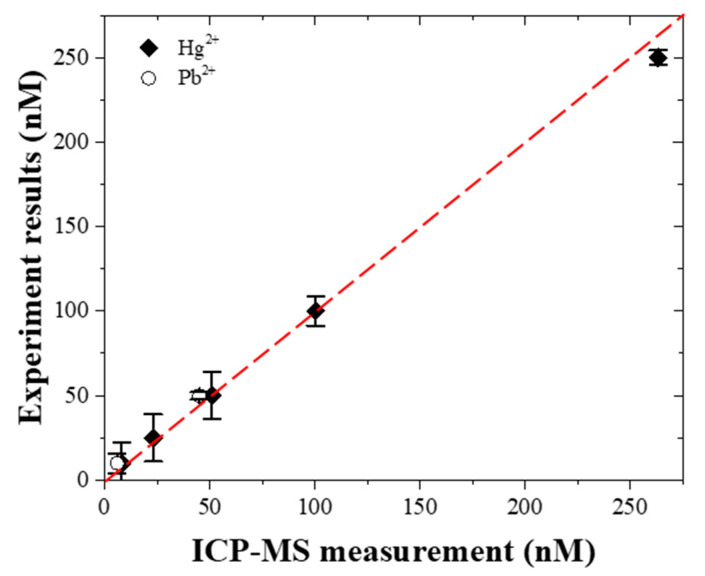
Comparison of results obtained by the ICP-MS and the present microfluidic device (*n* = 5).

The results show that the fluorescence intensity increases linearly with an increasing Hg^2+^ and Pb^2+^ concentration over the ranges of 10~250 nM (i.e., 2.0~50 ppb) and 10~100 nM (i.e., 2.1~20.7 ppb), respectively. Moreover, the 3.3σ/slope was used to determine the limits of detection (LoD), where σ is the standard deviation of the response [53,54]. The LoD of Hg^2+^ and Pb^2+^ is equal to 0.70 ppb and 0.53 ppb, respectively. The results show that the sensing platform provides a feasible solution for enforcing the WHO recommendation of no more than 6 ppb Hg^2+^ and 10 ppb Pb^2+^ in drinking water. Finally, to verify the detected results obtained by our measurements, the results acquired by the Uni-President Enterprises Corporation at Food Safety Laboratory (ISO/EIC 17025, No. 1021152009 issued by the FDA, Tainan, Taiwan) using ICP-MS were compared. Figure 6 is a standard comparison of the detected mean value between two independent laboratories. It shows excellent agreement between the two results.

### 3.3. Selectivity of Hg^2+^ and Pb^2+^ Detection

**Figure 7:**
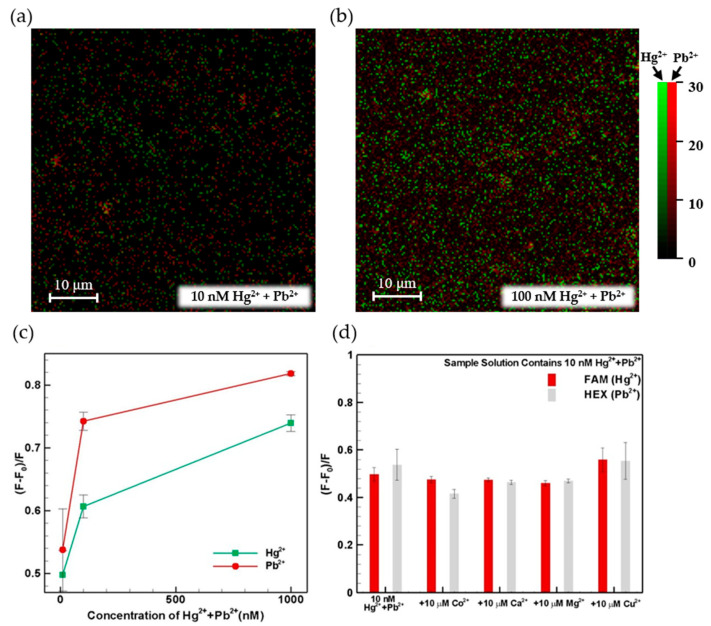
Fluorescence image of detecting Hg^2+^ and Pb^2+^ ions simultaneously for the sample Hg^2+^ and Pb^2+^ under (**a**) 10 nM and (**b**) 100 nM, respectively. Fluorescence intensity recovery for competitive assays involving: (**c**) different concentrations of Hg^2+^ and Pb^2+^, and (**d**) 10 μM Co^2+^, Ca^2+^, Mg^2+^ and Cu^2+^, respectively (500 ppm GO).

To verify the selectivity of the proposed aptamer detecting the Hg^2+^ and Pb^2+^ ions simultaneously, the GO/aptamer (GO + FAM(Hg^2+^) + HEX(Pb^2+^)) solutions were injected into the lower inlet of the microfluidic device, while a mixture of the Hg^2+^ and Pb^2+^ samples with concentrations of 10, 100 and 1000 nM, respectively, was injected into the upper inlet. Figure 7a,b show the detected fluorescence images for Hg^2+^ and Pb^2+^ samples of 10 nM and 100 nM, respectively. The fluorescence images reveal that the proposed aptamer is able to detect both ions simultaneously, and the fluorescence intensity is proportional to the concentration. The corresponding fluorescence intensity measurements are shown in Figure 7c. For both probes, the fluorescence intensity shows good recovery performance for all values of the concentration (i.e., low, medium and high). In other words, even though the fluorescence recovery reduces as the concentration of the competing metal ions increases, the device still retains the ability to discriminate the target ions. In addition, control experiments were performed using several other bivalent metal ions, namely Co^2+^, Ca^2+^, Mg^2+^ and Cu^2+^ ions. Figure 7d shows the detection results obtained for a mixture of the Hg^2+^ + Pb^2+^ ions containing an additional 10 μM of Co^2+^, Ca^2+^, Mg^2+^ and Cu^2+^ ions, respectively. It can be seen that the competing ions have only a minor effect on the measured fluorescence change despite their relatively high concentration. Notice that our concentration of Hg^2+^ and Pb^2+^ is 10 nM, which is much more diluted than those of other metal solutions (10 μM), i.e., a 1000-times difference in concentration. Therefore, the intensity of Hg^2+^ and Pb^2+^ is not significantly different from other solutions. In other words, the selectivity of the proposed sensor for Hg^2+^ and Pb^2+^ ion detection is further confirmed.

## 4. Conclusions

This study proposes two FRET-based GO/aptamer sensors for the detection of Hg^2+^ and Pb^2+^ ions, respectively, in a PDMS microfluidic device. In the proposed approach, two specifically chosen aptamer sequences are labeled with FAM and HEX fluorescent dye for visualization purposes and are then quenched by GO. Following interaction between the labeled aptamers and the Hg^2+^ and Pb^2+^ ions, respectively, a discernable restoration of the fluorescent intensity occurs as a result of the FRET effect. The change in the fluorescent intensity is then used to inversely derive the corresponding Hg^2+^ and Pb^2+^ concentration. Sensing experiments were performed using Hg^2+^ and Pb^2+^ solutions with concentrations of 10, 25, 50, 100, 250, 500, 1000 and 2000 nM (i.e., 2~400 ppb), respectively. The results show that the fluorescence intensity increases linearly with an increasing Hg^2+^ and Pb^2+^ concentration over the ranges of 10~250 nM (i.e., 2.0~50 ppb) and 10~100 nM (i.e., 2.1~20.7 ppb), respectively. Moreover, the limits of detection (LoDs) for Hg^2+^ and Pb^2+^ are equal to 0.70 ppb and 0.53 ppb, respectively. Finally, the experimental results obtained for competitive assays involving Hg^2+^ and Pb^2+^ ions and other metal ions (Co^2+^, Ca^2+^, Mg^2+^ and Cu^2+^) show that the proposed device has good selectivity. Overall, the results show that the proposed sensing device and platform provide a feasible solution for enforcing the WHO recommendation of no more than 6 ppb Hg^2+^ and 10 ppb Pb^2+^ in drinking water.

## Data Availability

Data will be provided via requests to the corresponding author.
